# Deep Convolutional Generative Adversarial Networks to Enhance Artificial Intelligence in Healthcare: A Skin Cancer Application

**DOI:** 10.3390/s22166145

**Published:** 2022-08-17

**Authors:** Marco La Salvia, Emanuele Torti, Raquel Leon, Himar Fabelo, Samuel Ortega, Beatriz Martinez-Vega, Gustavo M. Callico, Francesco Leporati

**Affiliations:** 1 Department of Electrical, Computer and Biomedical Engineering, University of Pavia, 27100 Pavia, Italy; 2 Research Institute for Applied Microelectronics (IUMA), University of Las Palmas de Gran Canaria (ULPGC), 35001 Las Palmas de Gran Canaria, Spain; 3 Norwegian Institute of Food, Fisheries and Aquaculture Research (Nofima), 6122 Tromsø, Norway

**Keywords:** deep learning, hyperspectral imaging, medical hyperspectral images, synthetic data generation, deep convolutional generative adversarial networks

## Abstract

In recent years, researchers designed several artificial intelligence solutions for healthcare applications, which usually evolved into functional solutions for clinical practice. Furthermore, deep learning (DL) methods are well-suited to process the broad amounts of data acquired by wearable devices, smartphones, and other sensors employed in different medical domains. Conceived to serve the role of diagnostic tool and surgical guidance, hyperspectral images emerged as a non-contact, non-ionizing, and label-free technology. However, the lack of large datasets to efficiently train the models limits DL applications in the medical field. Hence, its usage with hyperspectral images is still at an early stage. We propose a deep convolutional generative adversarial network to generate synthetic hyperspectral images of epidermal lesions, targeting skin cancer diagnosis, and overcome small-sized datasets challenges to train DL architectures. Experimental results show the effectiveness of the proposed framework, capable of generating synthetic data to train DL classifiers.

## 1. Introduction

Artificial intelligence (AI) was firstly adopted in medicine in the 1980s [1]. However, only recently have researchers proposed solutions for the clinical practice. Capable of acquiring a broad mixture of information, wearable systems and modern sensors produce an astounding amount of data to train intelligent systems.

When associated with statistically complete and labeled datasets to be trained with, AI algorithms produce robust and reliable classification performance. Indeed, machine learning (ML) algorithm performance is directly proportional to the amount of training data available [2]. Nonetheless, the amount of labeled data is not usually sufficient in healthcare applications, particularly when researchers consider deep learning (DL) architectures. Thus, they focus on techniques to generate statistically relevant synthetic data that are representative of real situations [3]. Moreover, different studies proposed architectures which employ either traditional RGB (red, green, and blue) images [4,5], chest X-rays [6], electrocardiograms [7], or hyperspectral (HS) data [8] for diagnostic purposes. The latter enables precise clustering of tumors, providing affordable diagnosis [9] and a powerful guidance tool for surgical procedures [10]. In these works, the authors exploited traditional ML algorithms due to the poor dataset size.

Synthetic HS data could be generated by a mathematical model which would consider the interaction between light and matter. However, such solution is not feasible to be developed due to the physical uncertainties together with the computational complexity required to model those physical light–matter interactions.

The so-called data augmentation process [11] refers to either affine transformation, namely, either geometrical, color-based, or additive statistical-based noise. Hence, the procedure transforms the images to yield new samples and increase the statistical variability of the information contained in a dataset. Nonetheless, the size of the original data population limits the data augmentation usage. Indeed, it is not always possible to generate a suitable number of new samples as both the dataset and the number of augmentations are finite.

Researchers overcame such limitations by conceiving generative adversarial networks (GANs), a novel data augmentation methodology proposed in 2014 [12]. GANs comprise two networks competing in an adversarial game based on game theory. The former, called *generator*, produces data whose distribution relates to the statistical distribution of the training samples. The latter, called *discriminator*, determines whether the input data belong to the real distribution.

Concerning healthcare applications [13], authors already adopted GANs in image denoising [14], segmentation [15], classification [16], and image synthesis [17]. Nonetheless, the application of GANs to hyperspectral imaging (HSI) is still at early stages since, to the best of the authors’ knowledge, only a preliminary study is available [7]. Indeed, it only introduces a proof of concept, proving the capability of GANs to generate HS skin cancer images. The authors validated their results only by comparing the typical average spectral reflectance of real and synthetic data. However, this research suffers several limitations [8]. Despite that the authors considered four different lesions, namely, dysplastic nevus, melanoma in situ, malignant melanoma, and benign nevus, they conceived a final validation concerning a typical spectral reflectance without comparing the different lesions. Moreover, the GAN-generated image class is unknown.

In this paper, we propose a deep convolutional GAN (DCGAN) to generate synthetic HS epidermal lesion images employing a small-sized dataset. Hence, not only did we validate the final generative model by using the synthetic data to train a ResNet18, which in turn is used to classify the original real training data, but we also evaluated the performances in terms of the Frechèt inception distance (FID) [18], accuracy, precision, recall, and *F*1 *score*.

In particular, the novel contributions proposed by this paper are as follows: (1) a DCGAN architecture extended to generate synthetic hyperspectral medical images; (2) the adoption of state-of-the-art techniques such as transfer learning and label smoothing; (3) the modification of the proposed DCGAN into a conditional network; (4) the use of a ResNet18 network to evaluate the similarity between synthetic and real datasets.

The paper is organized as follows: Section 2 describes the training dataset and the architecture of the proposed DCGAN. Furthermore, we introduce the training method and the evaluation metrics. Section 3 describes the performed experiments and the obtained results. Finally, Section 4 draws the conclusions of the proposed work.

## 2. Materials and Methods

### 2.1. Hyperspectral Skin Cancer Images Dataset

**Figure 1:**
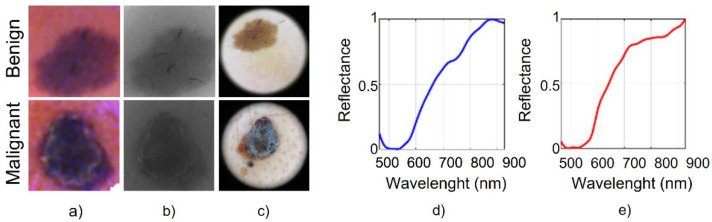
Example images from the dataset. (**a**) Pseudo-RGB images obtained with the HS camera, (**b**) grayscale image captured with the monochromatic sensor, (**c**) RGB images obtained with the digital dermoscopic camera, (**d**) average spectral signature of benign lesion, (**e**) average spectral signature of malignant lesion.

We considered a medical HS in vivo dataset [9] which consists of 76 HS images, 40 benign and 36 malignant skin lesions, taken from different body parts from 61 subjects. The data acquisition campaign was carried out from March 2018 to June 2019 at two hospitals: Hospital Universitario de Gran Canaria Doctor Negrín (Canary Islands, Spain) and the Complejo Hospitalario Universitario Insular-Materno Infantil (Canary Islands, Spain) [19]. The study protocol and consent procedures were approved by the Comité Ético de Investigación Clínica-Comité de Ética en la Investigación (CEIC/CEI) from both hospitals and written informed consent was obtained from all subjects. The acquisition system is composed of an HS snapshot camera (Cubert UHD 185, Cubert GmbH, Ulm, Germany) in the VNIR (visual and near-infrared) range, coupled to a Cinegon 1.9/10 (Schneider Optics Inc., Hauppauge, NY, USA) lens with a F-number of 1.9 and a focal length of 10.4 nm. The illumination system employs a halogen light source (150 W) coupled to a fiber optic ring light guide for cold light emission. The lighting system and HS camera are attached to a dermoscopic lens using a customized 3D printed part. Such dermoscopic lens allows direct contact with the skin, since it has the same refraction index as the human skin. Each image has a spatial resolution of 50 × 50 pixels and a spectral resolution of 8 nm, covering 125 spectral bands ranging from 450 to 950 nm (Figure 1a shows two synthetic images as examples). The HS also integrates a monochromatic sensor capable of capturing the same scene with a conventional monochromatic image with a resolution of 1000 × 1000 pixels (Figure 1b). In addition to the HS image and the monochromatic image, conventional RGB images of 3000 × 4000 pixels of the same skin lesion were captured (Figure 1c) using a standard digital dermoscopic camera (3Gen Dermlite Dermatoscope, 3Gen Inc., San Juan Capistrano, CA, USA). The illumination system employs a halogen source light (150 W) coupled to a fiber optic ring light guide for cold light emission. HS images were preprocessed and calibrated with white and dark references to standardize the spectral signatures [19]. In addition, since the first five and the last four bands contained high noise, we removed them from the images, having a final size of 50 × 50 pixels and 116 bands, covering an effective area of 12 mm × 12 mm. The acquisition time of the system is less than 1 s.

Dermatologists diagnosed the skin cancer and a pathologist performed a biopsy-proven histological assessment of suspicious lesions to obtain the definitive diagnosis. We performed the manual segmentation and labeling of each HS image, and in the end, data were labeled into two different classes, namely, *Benign* and *Malignant* (Figure 1d,e). The procedure resulted in labels encoded in one-hot format. However, the literature reports one-hot encoding often entailing discriminator model overconfidence. Thus, we employed a label smoothing technique [20] to solve this issue. Namely, we assigned the positive class to malignant lesions, whilst we assigned benign ones with 0. Therefore, we replaced the positive class with a random value ranging from 0.7 to 1 and the other with stochastic values from 0 to 0.3.

### 2.2. Deep Convolutional Generative Adversarial Networks

**Figure 2:**
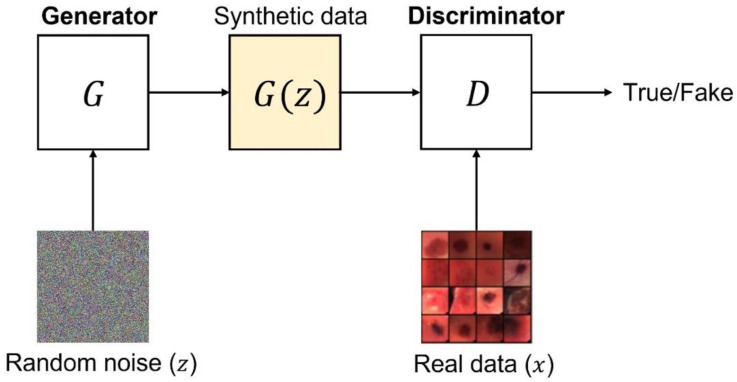
GAN standard structure.

The original GAN model was proposed in 2014 [12] and it was based on two subnetworks: a generator (G) and a discriminator (D). Figure 2 depicts the basic idea behind a GAN.

The generator G takes as input a latent space vector z from a standard Gaussian distribution and produces a sample G(z). This sample represents the mapping from the latent space z to the real data space. On the one hand, G is optimized to estimate the training data distribution and generate synthetic samples having the same distribution of the real data. On the other hand, the discriminator D receives as input the synthetic data produced by G or a sample (x) coming from the real dataset. D outputs the probability estimate concerning the input data source. Specifically, it estimates whether the sample came from the training data or from G. G and D play a minimax game, where G tries to minimize the probability that D will predict its outputs as fake, whilst D tries to maximize its probability to correctly discriminate between real and fake samples.

Researchers proposed several network architectural topologies to implement G and D [13], including Vanilla GAN [21], BiGAN [22], infoGAN [23], variational autoencoder network GAN (VAEGAN) [24], and deep convolutional GAN [25]. In recent years, deep convolutional neural networks have emerged as a stable and affordable architecture for synthetic image generation [26]. This architecture adopts two convolutional networks as G and D. In particular, G consists of transposed convolutional layers, while D is based on convolutional layers.

**Figure 3:**
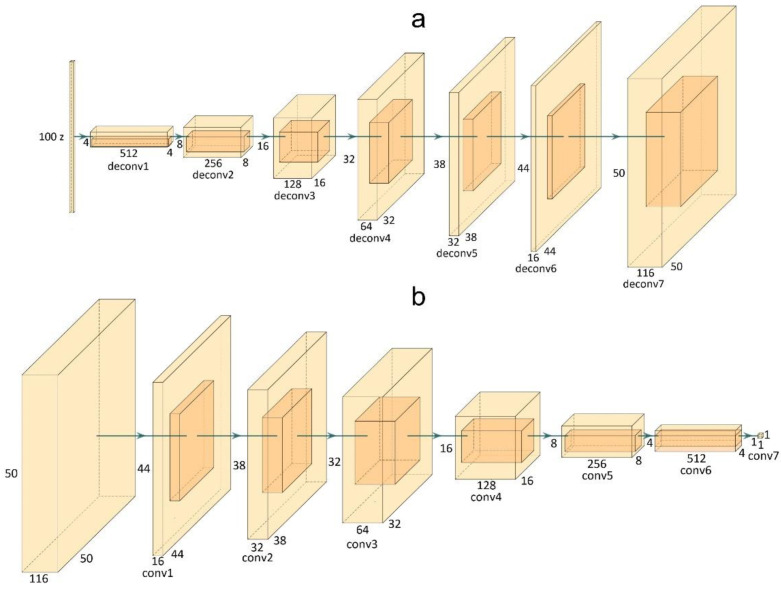
Proposed generator (**a**) and discriminator (**b**) architectures.

Considering HS images, the conversion from z to the data space performed by G consists of creating synthetic HS images with the same spatial and spectral dimensions of the training images. Since we employed as training set the skin cancer dataset described in Section 2.1, G should generate an image whose sizes are 50 × 50 × 116. Figure 3a shows the G architecture and the sizes adopted in this work for G. The deconvolutional layers from 1 to 6 are followed by a batch normalization and the ReLU activation function. Finally, the last deconvolutional layer adopts the *tanh* as activation function.

On the other hand, D receives as input an HS image with the same size, 50 × 50 × 116, and performs a binary classification to determine if the input image is real or fake. For this reason, this network is based on convolutional layers. Figure 3b depicts the architecture of D detailing the size of each convolutional layer. The first convolutional layer is characterized by the leaky ReLU activation function. The layers from 2 to 5 feature the batch normalization and the leaky ReLU activation function. All the leaky ReLU functions adopt a negative slope equal to 0.2. The final convolutional layer is characterized by the sigmoid function.

### 2.3. Transfer Learning

Authors who proposed GANs architectures [12] typically trained the framework adopting large datasets, such as CIFAR-10 [27], MNIST [28], or SVHN [29], which include 60,000, 70,000, and 600,000 images, respectively. It is worth noticing that the dimensionality of those datasets is huge when compared to the 76 images considered in this paper. This is a critical aspect addressed in this study to ensure that the generative model is capable of correctly approximating the original data distribution. Among the possible solutions, researchers usually adopt transfer learning to overcome the issue. It consists of using a model previously optimized for a task whose dataset size was bigger. It becomes the starting point to tackle a new problem, whose training set is smaller. In this context, the transfer learning approach consists of pretraining the GAN using RGB skin cancer images and using the obtained parameters as initialization for the final model, which is trained using the HS dataset. Thus, we trained the initial model using the HAM10000 dataset [30], randomly selecting 5000 RGB images from the database. We resized the images to 50 × 50 pixels to have the same HS dataset spatial dimension. Moreover, we modified the output layer of G and the input layer of D to address 3 channels instead of 116.

**Figure 4:**
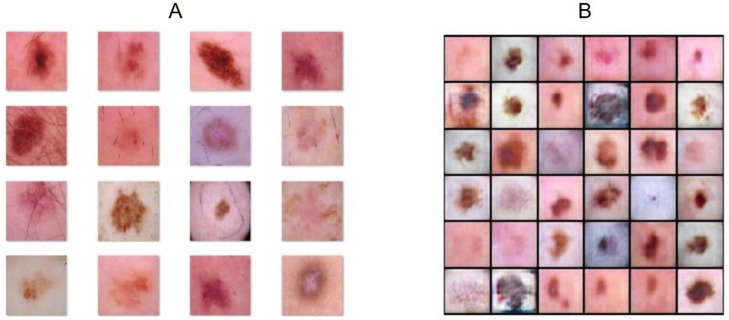
(**A**) Images taken from the training set. (**B**) Images generated by the architecture.

We adopted the Adam optimization method for the backpropagation algorithm, with learning rate set at 0.0002 for both networks, and a batch size of 128. All the hyperparameters were chosen adopting a trial-and-error approach, repeating the training phase with different values. The training elapsed after 100 epochs. Finally, we exploited a label swapping technique to avoid discriminator overfitting, which would imply no learning for the generator network. Figure 4 exhibits some images taken for the original dataset and different images generated by the network.

We transferred the network weights retrieved at the end of this training process to the architectures described in Figure 3. In particular, we only changed the output layer of G and the input layer of D. These layers had a size of 116; thus, the values obtained by the training with the RGB dataset were used to initialize the weights related to the channels associated to the green, red, and blue wavelengths. The remaining values were initialized in a pseudorandom way. In this phase, the batch size was reduced to 2. Moreover, we changed the size of the output layer of G from 116 to 117. The new channel is used to generate the segmentation mask related to the synthetic image. The mask generation is of critical importance since it includes information that can be used in the training process of a generic deep segmentation network, highlighting the lesion contours.

**Figure 5:**
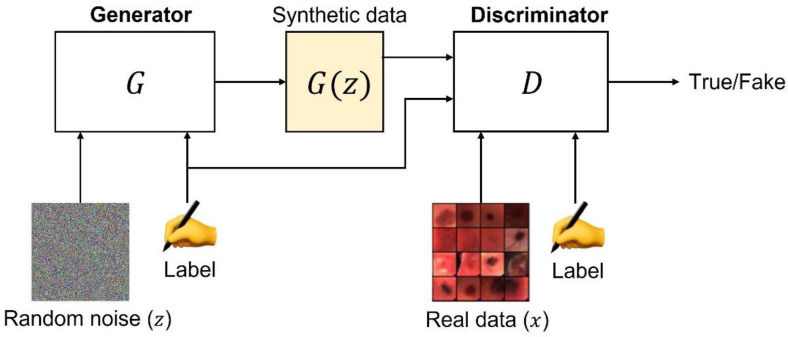
The cGAN architecture.

Finally, the proposed architecture was altered into a conditional GAN (cGAN). It means that G receives as input, together with the random noise vector, the class label-smoothed value which the synthetic image should belong to. Namely, the G can alternatively generate fake data related to the benign or malignant classes. The architecture of the proposed cGAN is shown in Figure 5.

We trained the cGAN for 200 epochs. During training, different methods were exploited to improve the quality of the synthetic images. The weights of each layer were scaled by a factor c according to the equalized learning rate rule [31]:

(1) $$c=\frac{\sqrt{2}}{\sqrt{input\_channels}}$$

where input_channels represents the number of input channels to the considered layer. Moreover, the two time-scale update rule (TTUR) was implemented [32]. Specifically, we assigned the two networks different learning rate values. The learning rate of G was lower than the one assigned to D. Thus, the weights related to G were updated with more steps than the ones assigned to D, to enhance the quality of the synthetic images.

To avoid D learning to discriminate real from fake images in a few training iterations, we swapped the labels for a random 5% of the training data. Indeed, we treated some fake images as real and vice versa. Finally, we adopted L2 regularization at $10^{-5}$ to reduce overfitting.

### 2.4. ResNet18 Classification

We employed a ResNet18 to measure real and synthetic HS data closeness. Namely, we trained the architecture only with synthetic HS images to classify the real epidermal lesions dataset. Therefore, we exploited overfitting as a measure to understand how well the synthetic data reproduces the real statistical distribution. This approach has not yet been proposed in the literature and was developed to reveal if the synthetic dataset represents a significative description of the real dataset. In this specific case, overfitting should not be seen as a negative effect. Indeed, overfitting on the synthetic dataset and obtaining good performance in the classification of the real dataset means that the obtained model generalized the considered problem. Results reported in Section 3 highlight the trustworthiness of our generated HSIs.

**Figure 6:**
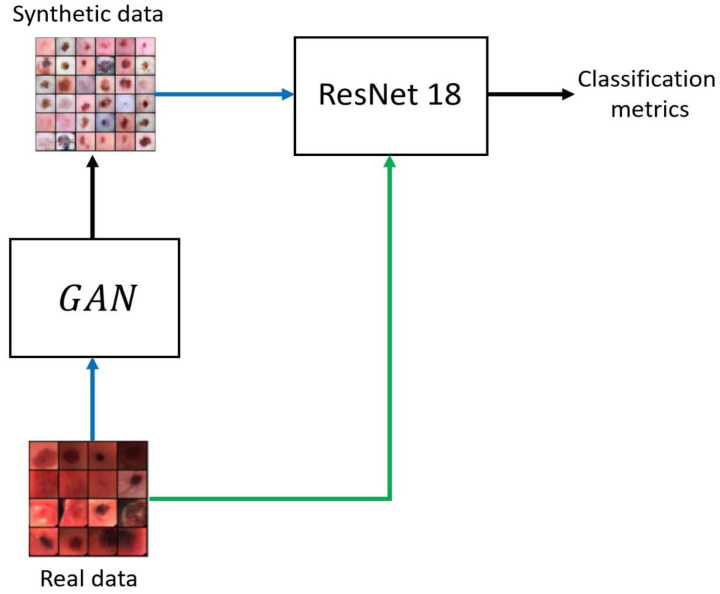
The proposed methodology to evaluate the similarity of the datasets. The blue arrows indicate that a set was used to train a model. The green arrow indicates that the set is classified by the network.

The proposed approach is depicted in Figure 6, where the blue arrows indicate that the set was used to train the model, while the green arrow denotes that the dataset is used as input for the classification.

The ResNet was pretrained on the ImageNet database, and then modified to classify the hyperspectral images. The pre-trained Resnet is available online (https://it.mathworks.com/help/deeplearning/ref/resnet18.html#mw_591a2746-7267-4890-8390-87ae4dc7204c_sep_mw_6dc28e13-2f10-44a4-9632-9b8d43b376fe (accessed on 10 July 2022)). The input layer was changed to consider as input an image of size 50 × 50 × 116. Moreover, the network was trained adopting the Adam gradient descent method considering 50 epochs. The ResNet was trained with 1000 synthetic images while the test set included only real images.

### 2.5. Evaluation Metrics

We employed several evaluation metrics for the measure of the performance of the developed generative framework. Frechèt inception distance (FID) is the state-of-the-art metric to assess the performance of a GAN in terms of quality of the synthetic images [18]. The FID metric calculates the distance between the calculated feature vector for the real image and the generated image. Thus, a low value ensures that the two sets are similar. The FID is defined as follows:

(2) $$FID=\|\mu_{1}-\mu_{2}{\|}_{2}^{2}+Tr\left(\Sigma_{1}+\Sigma_{2}-2\sqrt{\Sigma_{1}\Sigma_{2}}\right)$$

where μ represents the mean value, Σ is the covariance matrix and Tr indicates the trace of a matrix. The subscripts 1 and 2 indicate the real and the synthetic images sets, respectively.

Concerning the ResNet18 classification performance, we employed accuracy, precision, recall, and *F*1 *score*. Accuracy is defined by Equation (3), where *TP*, *TN*, *FP*, and *FN* are the number of true positives, true negatives, false positives, and false negatives, respectively. Precision indicates the true percentage of positive identification whilst recall reports the percentage of actual positives correctly identified, in Equations (4) and (5), respectively. The F1 score, shown in Equation (6), is the harmonic mean of precision and recall.

(3) $$accuracy=\frac{TP+TN}{TP+TN+FP+FN}$$

(4) $$precision=\frac{TP}{TP+FP}$$

(5) $$recall=\frac{TP}{TP+FN}$$

(6) $$F1\;score=2\cdot \frac{precision\;\cdot \;recall}{precision+recall}$$

## 3. Experimental Results

The synthetic images quality was evaluated in two ways. On the one hand, we employed a gold standard metric in GANs, the FID [18]. On the other hand, we evaluated the accuracy, precision, recall, and *F*1 *score* of a ResNet18, trained only with synthetic images, and then validated on the original dataset. Namely, we exploited overfitting to assess synthetic and real data distribution closeness. For these tests, the generator produced a total of 1000 synthetic HSIs of skin lesions, equally balanced between benign and malignant classes.

### 3.1. Frechèt Inception Distance

The synthetic HS dataset generated by G obtained an FID value of 17.37. To evaluate and compare different FID results, we computed the FID between the original data distribution and its augmented version. In particular, we simply horizontally flipped every HS image in the dataset. In this case, we measured an 8.96 FID value. It is worth noticing that the two FIDs are close, thus indicating that the synthetic and the real data are similar. The comparison between the two FIDs was performed to highlight that the value obtained by the proposed network indicates that the two sets are similar.

### 3.2. ResNet18 Classification Performance

We exploited the synthetic dataset to train a ResNet18 network to classify the real HS dataset. The ResNet18 is trained for 50 epochs with the 1000 generated synthetic images. The network achieved 100% accuracy on the training set, thus overfitting it. Furthermore, we used the architecture network to classify all the images included in the real dataset.

We report the performance obtained by the ResNet18 in the classification of the real images in Table 1.

Data reported in Table 1 clearly show that the ResNet18 is capable of correctly classifying most of the real images. Thus, these results indicate that the synthetic and the original dataset are similar. Moreover, we also trained the ResNet18 using only the real dataset and applying standard data augmentation techniques. The obtained results are closer to the values reported in Table 1. In particular, accuracy, precision, recall, and *F*1 *score* are 85.52%, 83.50%, 85.65%, and 92.77%, respectively. Nonetheless, it is worth noticing that the values should not be compared. The first results allow data leakage on purpose to assess the presence of overlap between the real and synthetic data distributions. On the other hand, the training on real data foresaw a train–test split to avoid the aforementioned data leakage and accurately assess generalization capabilities of the model on new data. In conclusion, the difference between the metrics in the two training scenarios highlights that the synthetic data quality might be further increased before its usage to enlarge the training set.

### 3.3. Spectral Signature Analysis

**Figure 7:**
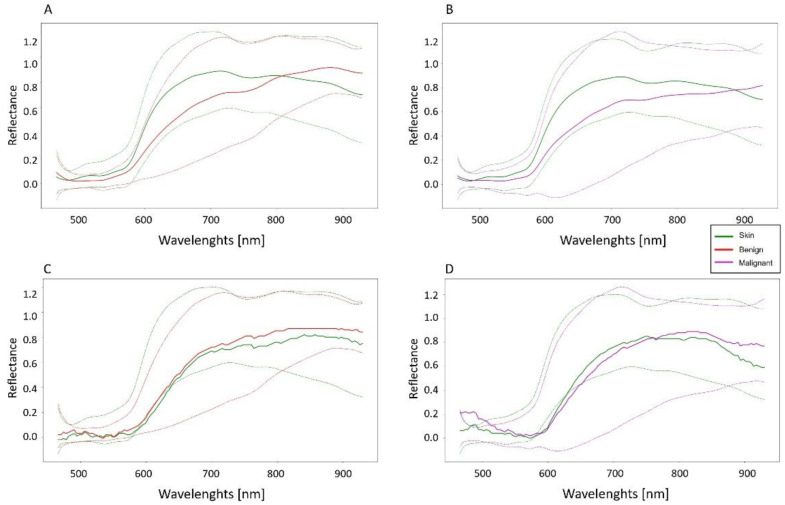
Comparisons between real and synthetic spectral signatures. (**A**,**B**) represent the real dataset spectral signatures, while (**C**,**D**) represent the synthetic (fake) ones after the smoothing operation. The dashed lines are twice the standard deviation ranges of the signatures, while the continuous lines represent the mean values.

The synthetic and the original datasets were also compared in terms of spectral signatures. Figure 7 displays the comparison between the original and the synthetic spectral signatures of the skin and the malignant and the benign lesions. From a visual inspection of the average spectral signatures and their ranges of variation, it is possible to observe that the synthetic data can be used to describe the same distribution of the original dataset.

A quantitative comparison between the spectral signatures can be carried out adopting the Jensen–Shannon divergence [33], given by (7):

(7) $$JS\left(v,w\right)=\frac{1}{2}\;\sum_{i}\left(v_{i}log\left(v_{i}\right)+w_{i}log\left(w_{i}\right)-\left(v_{i}+w_{i}\right)log\left(\frac{1}{2}\left(v_{i}+w_{i}\right)\right)\right)$$

where v and w are the spectral signature to compare, and i represents the i-th band.

The Jensen–Shannon divergence is equal to 0.6, 0.10, and 0.04 for the benign, malignant, and skin synthetic and real signatures, respectively. It is worth noticing that this metric is bounded by 1 for two distributions. Thus, the obtained values clearly highlight the similarity between the real and synthetic signatures.

### 3.4. Comparisons with the State-of-the-Art

Researchers widely explored GANs to generate synthetic images. However, the literature is focused on generating synthetic data that typically is not HS images. Thus, a fair comparison can only be made with the work reported in [8], which considered HS images related to skin cancer. The work reported the results only in terms of mean spectral signature of the whole synthetic dataset. No FID is computed between the real and the synthetic dataset.

These considerations highlight that the proposed research describes and analyzes, in a broader and more comprehensive way, a GAN architecture capable of generating hyperspectral synthetic data even if the training set includes a low number of examples.

### 3.5. Limits of the Proposed Approach and Future Development

Data-centric applications strongly rely on the dataset size, influenced by subjects participating in clinical research and data acquisition campaigns. The data availability challenge is exhibited in scenarios such as ours where physicians employ a novel, non-standardized, and uncommon technology in routine clinical practice. Moreover, data protection regulations currently obstruct research data sharing. Therefore, we proposed synthetic data assembling to overcome these limitations, providing researchers with increased and anonymous data [34], accelerating deep learning methodologies into general clinical practice [35]. In recent years, synthetic data generation has attracted considerable attention in the medical field, enhancing existing AI [36] with novel data augmentation methodologies. Nonetheless, experimenters must provide knowledge concerning synthetic and original data distributions [37]. Not only could the synthetic data be evaluated through quantitative appraisal, but it could also be evaluated with qualitative assessment processes provided by medical experts [3,38].

We engineered a proof-of-concept to produce synthetic data to enhance and accelerate the development of AI algorithms for a specific context, especially when scientists engage a limited HS dataset to engineer a decision support system to aid skin cancer diagnosis. We aim to pave the course for deep learning techniques in medicine when the number of labeled samples is limited. Nonetheless, investigators should carry out large data acquisition campaigns to include data from several subjects, including different skin lesion types and many clinical centers. Additionally, physicians should perform a rigorous clinical study to validate the usefulness of the offered solution. Dermatologists should evaluate whether the HS spatial information correlates with the morphological features belonging to the different skin lesions. Therefore, qualitative evaluations could assess the similarity between the original and synthetic skin lesions distributions through a heuristic blind evaluation test. Finally, scientists should evaluate several HS camera models to develop a generative instance capable of producing distinct data distributions.

## 4. Conclusions

This paper proposes a convolutional DCGAN architecture to generate HS medical data, particularly for skin lesion analysis. We employed a small-sized dataset to train the GAN framework. First, the GAN was trained with 5000 RGB images taken from the HAM10000 dataset, and then the transfer learning methodology was applied to train the adversarial framework with the HS images.

We adopted the FID metric to evaluate the similarity between the real and the synthetic data. We measured a 17.37 FID, which indicates good synthesis and similarity between the distributions of the two datasets.

Moreover, a ResNet18 was trained only on synthetic data to classify the real images. The accuracy, precision, recall, and *F*1 *score* were all above 80%, proving again that the synthetic data and the real images are comparable. Finally, the spectral signatures were compared both qualitatively and quantitatively.

The literature reports only one work considering medical HS data [8]. However, this work validated the results only in terms of visual similarity between mean spectral signature of real and generated images.

Future research lines will focus on the investigation of novel GAN architectures for medical HS images. Finally, the conditional GAN could be extended not only to generate benign or malignant lesions, but to produce different tumor etiologies.

## Figures and Tables

**Table 1:** ResNet18 real HS dataset classification performance.

Metric	Value [%]
accuracy	84.21
precision	81.57
recall	86.11
F1 score	83.77
